# A safe and simple procedure for laparoscopic hepatectomy with combined diaphragmatic resection

**DOI:** 10.1007/s13691-021-00506-x

**Published:** 2021-08-11

**Authors:** Keisuke Oyama, Shin Nakahira, Sakae Maeda, Akihiro Kitagawa, Yuki Ushimaru, Nobuyoshi Ohara, Yuichiro Miyake, Yoichi Makari, Ken Nakata, Junya Fujita

**Affiliations:** Department of Surgery, Sakai City Medical Center, 1-1-1 Ebarajicho, Nishi-Ku, Sakai City, Osaka 593-8304 Japan

**Keywords:** Laparoscopy, Hepatectomy, Diaphragm, Surgical technique

## Abstract

Diaphragmatic resection may be required beneath the diaphragm in some patients with liver tumors. Laparoscopic diaphragmatic resection is technically difficult to secure in the surgical field and in suturing. We report a case of successful laparoscopic hepatectomy with diaphragmatic resection. A 48-year-old man who underwent laparoscopic partial hepatectomy for liver metastasis of rectal cancer 20 months ago underwent surgery because of a new hepatic lesion that invaded the diaphragm. The patient was placed in the left hemilateral decubitus position. The liver and diaphragm attachment areas were encircled using hanging tape. Liver resection preceded diaphragmatic resection with the hanging tape in place. Two snake retractors were used to secure the surgical field for the inflow of CO_2_ into the pleural space after diaphragmatic resection. The defective part of the diaphragm was repaired using continuous or interrupted sutures. Both ends of the suture were tied with an absorbable suture clip without ligation. In laparoscopic liver resection with diaphragmatic resection, the range of diaphragmatic resection can be minimized by performing liver resection using the hanging method before diaphragmatic resection. The surgical field can be secured using snake retractors. Suturing with an absorbable suture clip is conveniently feasible.

**Supplementary Information:**

The online version contains supplementary material available at 10.1007/s13691-021-00506-x.

## Introduction

Laparoscopic liver resection has been proven to be a better alternative than open liver resection in patients with liver metastases of colorectal cancer based on perioperative and oncological outcomes [1]. Liver metastases are associated with 23% of colorectal cancers [2]. Some patients with subdiaphragmatic liver metastases of colorectal cancer require diaphragmatic resection; 3.3% of liver metastases from colorectal cancer required diaphragmatic resection [3]. It has been reported that liver resection combined with diaphragmatic resection resulted in good surgical and oncological outcomes [4]. In patients with liver metastases invading the diaphragm, diaphragmatic resection is required to obtain negative surgical margins. When laparoscopic diaphragmatic resection and suturing is performed, maintaining pneumoperitoneum is difficult due to the inflow of CO_2_ into the pleural space. Laparoscopic diaphragmatic suturing is technically difficult due to respiratory movement, narrow surgical field, and upside-down conditions. It is necessary to devise a technique to minimize the range of diaphragmatic resection and suture easily. We report a case in which laparoscopic hepatectomy with diaphragmatic resection was safely performed using a simple procedure.

## Case report

**Fig. 1:**
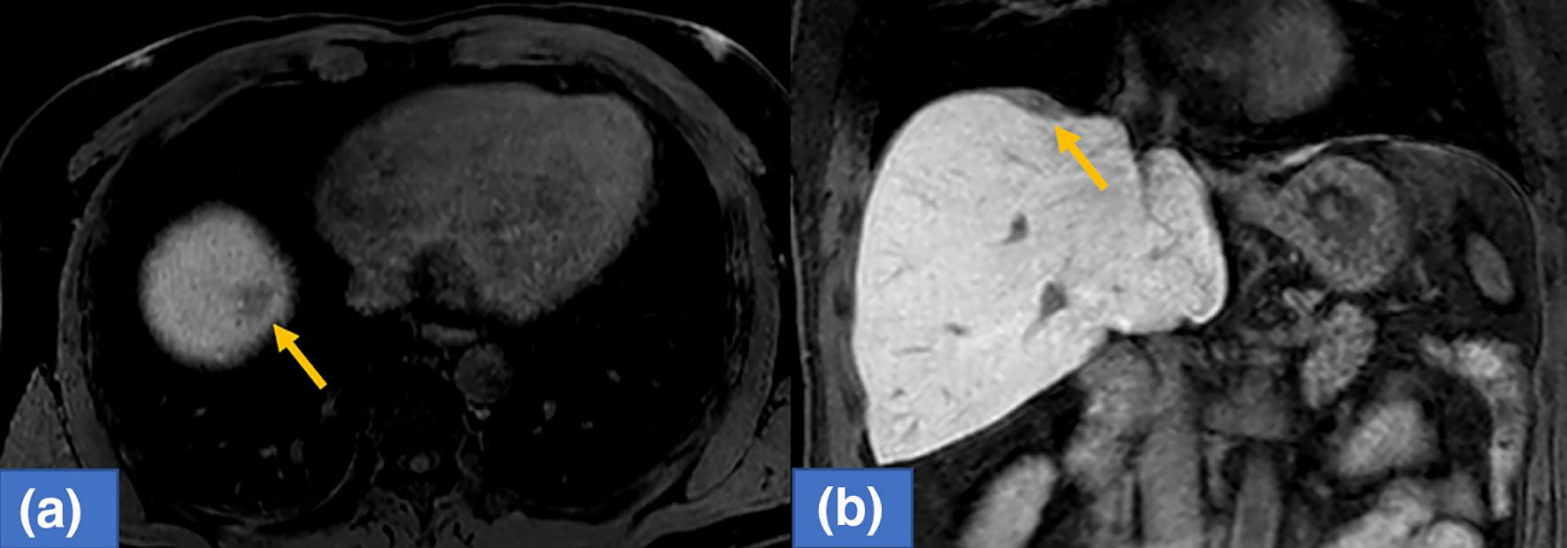
EOB-MRI revealed a hypointensity tumor located just below the diaphragm at liver segment 8 on hepatobiliary phase (arrow). (**a**: axial sections, **b**: coronal sections)

**Fig. 2:**
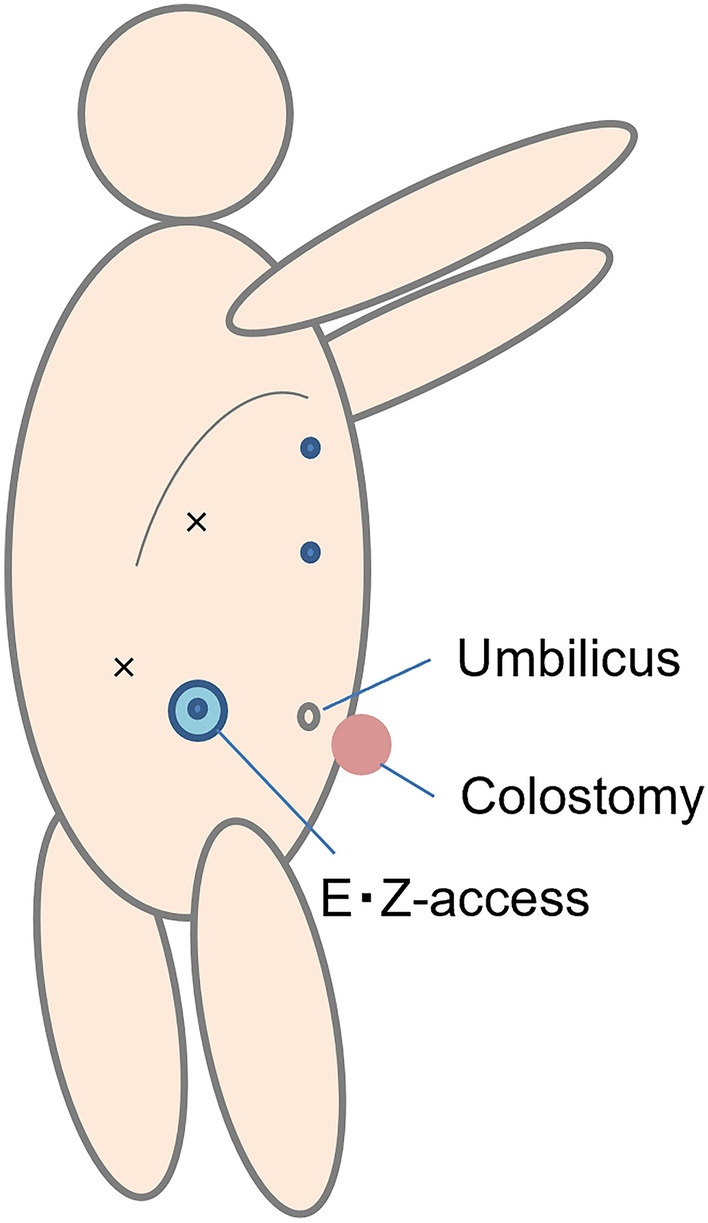
The illustration of the patient and port placement. The patient was placed in the left hemilateral decubitus position. EZ-access was inserted through the right lateral umbilical. Circles represent 12-mm and crosses represent 5-mm ports. 12-mm EZ-access, 12-mm epigastric, 12-mm upper abdominal, 5-mm right subcostal and 5-mm right lateral ports were inserted

**Fig. 3:**
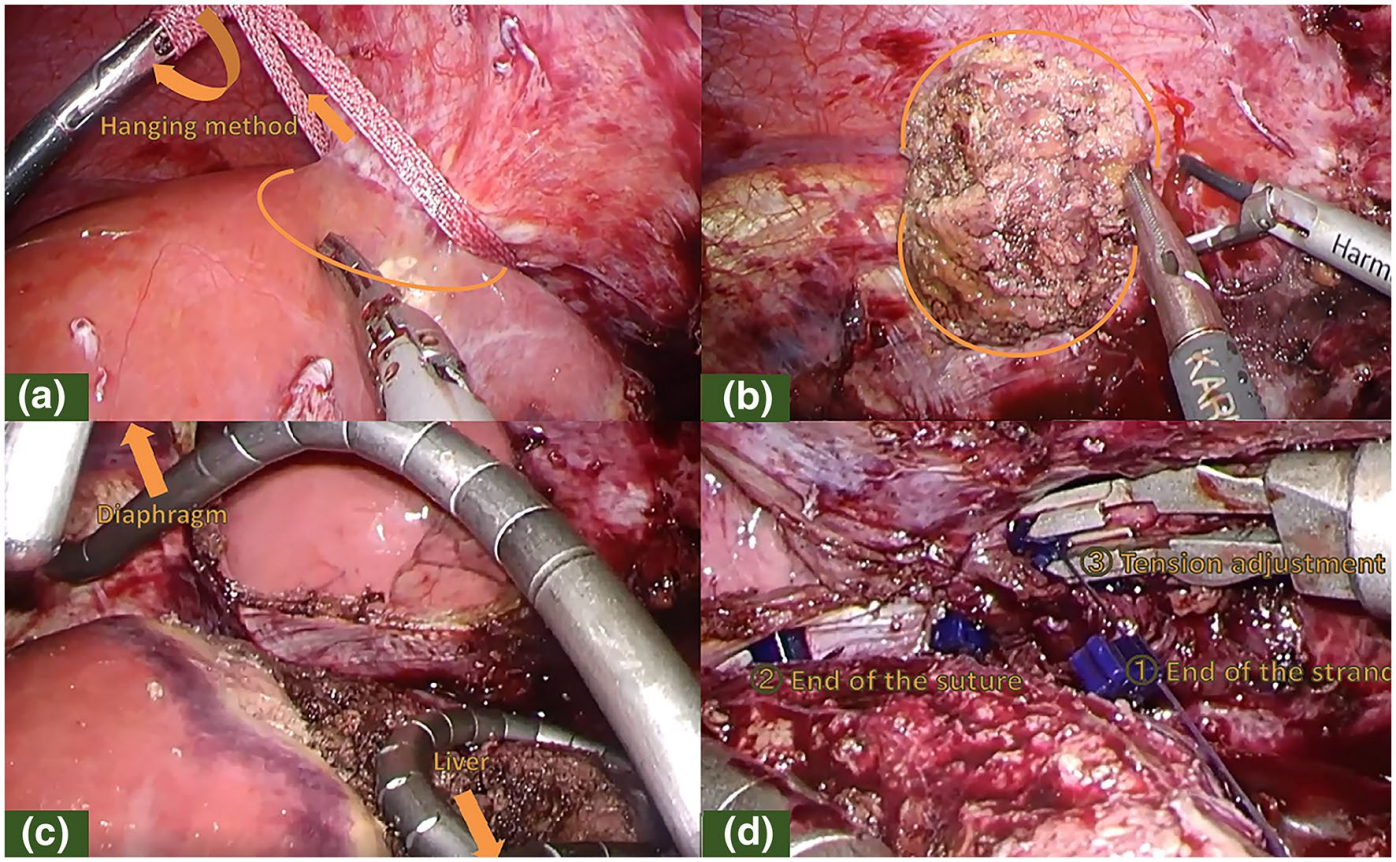
Surgical image. **a** Liver resection using the hanging method prior to diaphragmatic resection. **b** Diaphragmatic resection while confirming the invading range of the tumor. **c** Securement the surgical field using two snake retractors. **d** Repairing the diaphragm with LAPRA-TY® clips. ①Attaching LAPRA-TY® clips to the end of the string. ②Attaching the end of the continuous sutures. ③Adjusted to the tension of the diaphragmatic sutures

**Fig. 4:**
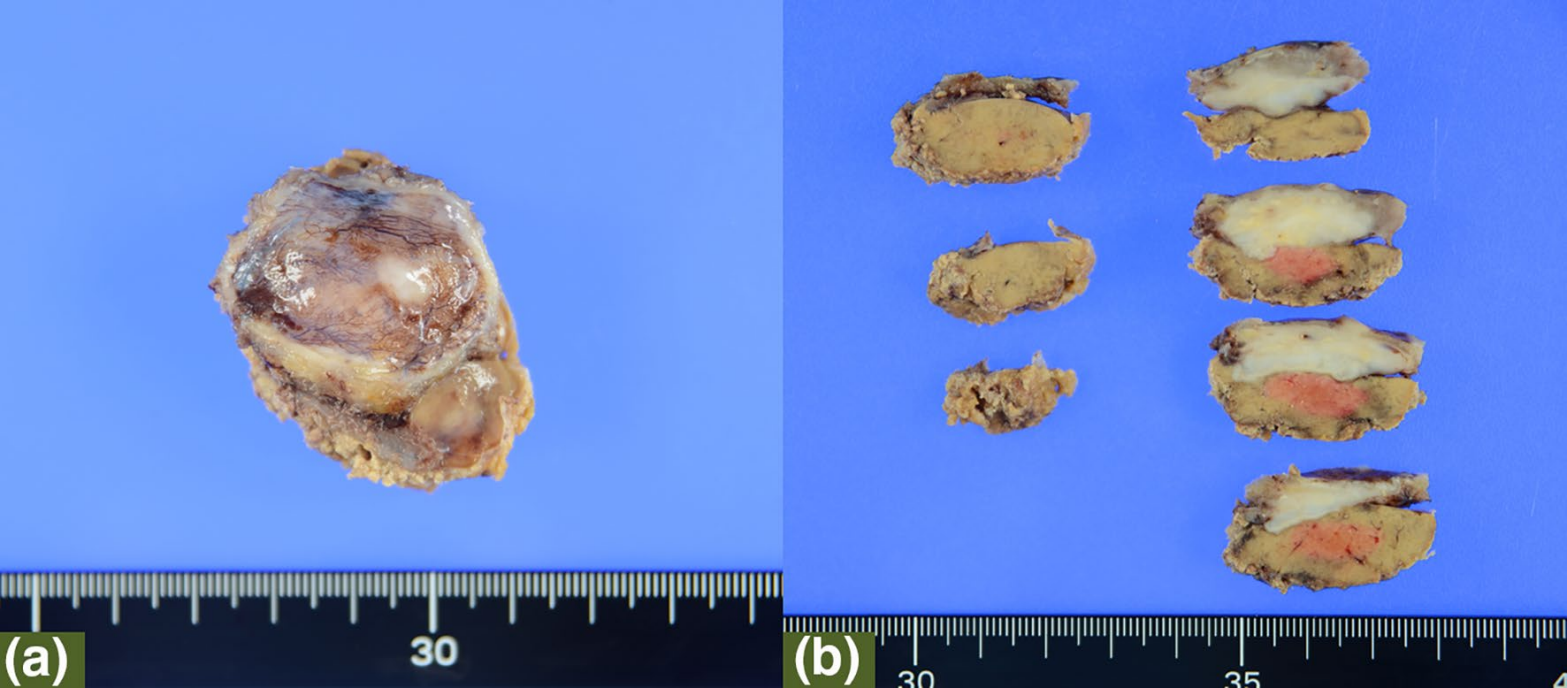
**a** The surgical specimen. The pathological diagnosis was liver metastasis of rectal cancer. The weight of the resected liver including the diaphragm was 5 g. The excision range of the diaphragm was 34 × 44 mm. **b** The cutting surface of specimen. The tumor had invaded the diaphragm. The surgical margins of the tumor were negative

A 48-year-old man underwent laparoscopic partial liver resection of segment 8 for liver metastasis of rectal cancer. Laparoscopic lower anterior resection was performed in patients with stage T3 rectal cancer. The pathological diagnosis was adenocarcinoma with no lymph node metastasis and the surgical margins of the tumor were negative. Twenty months after surgery, computed tomography revealed a new lesion beneath the diaphragm. Gadolinium ethoxybenzyl diethylenetriamine pentaacetic acid-enhanced magnetic resonance imaging (EOB-MRI) revealed a hypointense tumor located just beneath the diaphragm at the liver segment 8 on the hepatobiliary phase (Fig. 1). The surgery was performed due to growth in tumor size. The patient was placed in a left hemilateral decubitus position, and five laparoscopic ports were placed as described in Fig. 2. Pneumoperitoneum was established through the right lateral umbilical 12-mm camera port inserted using the open-entry method. Adhesions from the previous surgery were dissected. Tumor invasion to the diaphragm was detected while the right lobe was mobilized. The invaded area of the diaphragm was encircled using a hanging tape. Liver resection preceded diaphragmatic resection. Due to severe adhesions around the hepatoduodenal ligament and the small extent of resection volume, liver resection was performed without the Pringle maneuver. Liver resection was performed using HARMONIC ACE® (Ethicon Endo-Surgery, Cincinnati, OH, USA) and CUSA® (Clarity Ultrasonic Surgical Aspirator System, Integra LifeSciences Corporation, NR Ireland Limited) while hanging the liver tumor with tape. The inflow of CO_2_ into the pleural space after diaphragmatic resection created a poor surgical field due to the development of unsustainable pneumoperitoneum. The liver and diaphragm were held down using two snake retractors to secure the surgical field. The diaphragm defect was repaired with continuous or interrupted sutures placed using LAPRA-TY® (Ethicon Endo-Surgery, Cincinnati, OH, USA) clips and 3–0 Vicryl sutures. Both ends of the string were tied using a LAPRA-TY® clip without ligation. Continuous or interrupted sutures were placed based on the tension of the diaphragm while reducing the pneumoperitoneum pressure from 10 to 6 mmHg, and respiration was temporarily stopped while consulting with the anesthesiologist. The surgery was completed with a 14-French chest tube insertion. The patient had no respiratory or circulatory abnormalities during the surgery. The total operative time was 272 min with a 10-mL blood loss. The image of the surgical method is shown in Fig. 3. The video of the surgery is available online only (Supporting Information). The postoperative course was uneventful, the chest tube was removed after confirming no air leakage on the first postoperative day, and the patient was discharged 8 days after the surgery. The pathological diagnosis was liver metastasis of rectal cancer invading the diaphragm. The surgical margins of the tumor were negative. The tumor weighed 5 g, including the diaphragm. The excision range of the diaphragm was 34 × 44 mm (Fig. 4). Eighteen months after the surgery, no abnormalities were found in the diaphragm regarding respiratory symptoms and imaging findings. Written informed consent was obtained from the patient to publish this video article and any accompanying images presented in this study, and patient anonymity was preserved.

## Discussion

In repairing the diaphragm, primary suture or mesh placement is used based on the defect range. Reconstruction with pedunculated muscle flaps such as the external oblique muscle has also been reported [5]. Excessive tension in the diaphragm repair can lead to a risk of anastomotic leakage and adverse respiratory effects. Placing a mesh for diaphragm repair can also cause postoperative infections and pain. In laparoscopic hepatectomy with diaphragmatic resection, it is necessary to minimize the range of diaphragmatic resection. However, in the case of diaphragmatic resection before liver resection, the liver can be easily mobilized; the diaphragmatic resection cannot be performed while confirming the tumor invasion area into the diaphragm in laparoscopic hepatectomy with diaphragmatic resection. Minimal resection can be performed while confirming the range of diaphragmatic invasion by performing liver resection before diaphragmatic resection. When hepatectomy precedes diaphragmatic resection, the mobility of the liver may be restricted because the diaphragm and liver are fixed. By encircling the invaded area of the diaphragm with tape and hanging the liver tumor, even if the liver is not mobilized freely, the hanging tape can be pulled and rolled up to control the transection plane and maintain a good tension. A thoracoscopic approach may be useful in cases of repeat hepatectomy and lesions beneath the diaphragm [6]; however, our procedure is considered superior in terms of confirming the invading range of the diaphragm and minimizing the resection range. Following diaphragmatic resection, the inflow of CO_2_ into the pleural space creates a poor surgical field due to the development of unsustainable pneumoperitoneum. It is necessary to devise surgical field expansion and suturing in a state where the pneumoperitoneum cannot be maintained. In previous reports, pleural air was evacuated by a transthoracic catheter or a suction tip introduced via laparoscopic trocar to secure the surgical field [4, 7]. In our case, the surgical field can be secured using snake retractors. Snake retractors can be inserted through EZ-access® (Hakko Medical Co, Nagano, Japan) and lateral 5-mm ports. The bent shape permits surgical field deployment without interference with the other forceps. The surgical field can be easily secured without any invasion into the pleural cavity using a snake retractor. It is also necessary to devise a suitable procedure for diaphragm repair due to the difficulties of laparoscopic diaphragm sutures. The diaphragm tension could reduce the pressure exerted by the pneumoperitoneum, thereby facilitating suturing [8]. Suturing with continuous or interrupted sutures using an absorbable suture clip according to the tension of the diaphragm is conveniently feasible. An absorbable suture clip can be sutured without ligation by attaching it to the end of the string. There are cases in which LAPRA-TY® clips are used in diaphragmatic injury [9], which can significantly reduce technical difficulties. Non-absorbable sutures cannot be used when using an absorbable suture clip. Non-absorbable sutures are recommended for patients with diaphragmatic injuries and diaphragmatic hernias [10]. On the other hand, it is reported that the suture method should be selected according to the grade of diaphragmatic injury. Primary sutures with absorbable suture material can be performed if the diaphragm defect is within 10 cm [11, 12]. There were no reports of anastomotic leakage using absorbable sutures in patients who underwent hepatectomy with diaphragmatic resection. Moreover, no short or medium-term complications were observed in our patient. If the range of diaphragmatic resection is minimized as in this procedure, no problems may arise from suturing with an absorbable suture clip and using absorbable sutures. In this case, 3–0 Vicryl was used because the diaphragmatic resection was minimized and the tension was not strong. However, in cases of high abdominal pressure such as obesity or when the resection range is close to 10 cm, 2–0 Vicryl or thicker sutures and two-layer sutures are recommended. The 2–0 Vicryl can be attached with LAPRA-TY® clips, and the needle can be taken in and out through the EZ-access®. We found no reports on respiratory and circulatory abnormalities in patients who underwent laparoscopic diaphragmatic resection; however, there are existing reports on tension pneumothorax due to intraoperative diaphragmatic injury [13]. The laparoscopic surgical procedure should be stopped immediately if respiratory and circulatory abnormalities occur. Moreover, safe surgery is possible by temporarily stopping respiration when resecting or suturing the diaphragm. Cooperation with the anesthesiologist is especially crucial when performing laparoscopic diaphragmatic resection. We performed laparoscopic hepatectomy with diaphragmatic resection in a patient with liver metastasis of colorectal cancer, and the diaphragmatic resection was safely performed using a simple procedure.

## Supplementary Information

The video of the surgery is summarized in 96 seconds and introduces the surgical procedure of laparoscopic hepatectomy, diaphragmatic resection, and repair.

Below is the link to the electronic supplementary material.

Supplementary file1 (MP4 72270 kb)

## Abbreviations

EOB-MRI: Gadolinium ethoxybenzyl diethylenetriamine pentaacetic acid-Enhanced magnetic resonance imaging
